# Oxytetracycline as an otolith chemical marker in Atlantic bluefin tuna (*Thunnus thynnus*): Technical feasibility in culture

**DOI:** 10.1111/jfb.70516

**Published:** 2026-05-30

**Authors:** Inma Salvat‐Leal, Aurelio Ortega, Diego Romero, Edurne Blanco

**Affiliations:** ^1^ Área de Toxicología. Facultad de Veterinaria Universidad de Murcia, Campus Regional de Excelencia Internacional Mare Nostrum Murcia Spain; ^2^ CN‐IEO‐CSIC Instituto Español de Oceanografía, Centro Oceanográfico de Murcia Murcia Spain; ^3^ CN‐IEO‐CSIC Instituto Español de Oceanografía, Centre Oceanogràfic de les Balears Palma de Mallorca Spain

**Keywords:** aquaculture, chemical marking, otoliths, oxytetracycline, *Thunnus thynnus*, traceability

## Abstract

Reliable discrimination between wild and cultured fish is essential for traceability in aquaculture. This study reports the first successful application of intramuscular oxytetracycline (OTC) injection for otolith marking in juveniles of Atlantic bluefin tuna (*Thunnus thynnus*). Fifteen individuals (initial weight ≈ 100 g) were injected with either 100 mg kg^−1^ (*n* = 8) or 200 mg kg^−1^ (*n* = 7) OTC. All otoliths examined exhibited clear and persistent fluorescent bands under ultraviolet light, from 24 h up to 98 days post‐injection, with no significant differences between concentrations. These preliminary results indicate that intramuscular OTC injection is technically feasible as an otolith‐marking method in juveniles of this species.

Traceability and cohort differentiation remain significant challenges in the sustainable management of Atlantic bluefin tuna (*Thunnus thynnus*, ABFT). Although several chemical marking approaches have been effective in other species (e.g., immersion in marking solutions or the use of enriched feeds), the large body mass of ABFT, the volumes required and the dynamic nature of sea‐cage culture systems make these methods impractical.

Among chemical markers, oxytetracycline (OTC) has been used as a fluorochrome in fisheries research since the 1960s (Weber & Ridgway, 1962), as it incorporates into calcified structures within a few hours (Nagięć et al., 1995). In otoliths, OTC produces well‐defined fluorescent bands under ultraviolet light (Brooks et al., 1994; James et al., 2024; Warren‐Myers et al., 2018; Weber & Ridgway, 1967). Although intramuscular administration of tetracycline has been shown to be effective in several species of the family Scombridae (Adam et al., 1996; Foreman, 1996; Hallier et al., 2005; Krusic‐Golub & Ailloud, 2022; Sardenne et al., 2015; Wild & Foreman, 1980), its applicability in *T. thynnus* has not been documented. In this context, we evaluated whether intramuscular OTC injection could effectively mark the otoliths of captive‐born juvenile ABFT.

Fifteen juvenile ABFT (mean weight ≈ 100 g), produced from natural spawning in sea cages (San Pedro del Pinatar, Spain), were reared at the Spanish Institute of Oceanography CSIC facilities in Mazarrón following standard protocols (see Salvat‐Leal et al., 2023). At 42 days post‐hatch (dph), the fish were transferred to a 55‐ m^3^ overflow tank and assigned to two treatment groups: 100 mg kg^−1^ (*n* = 8) and 200 mg kg^−1^ of OTC hydrochloride (*Acofarma*, Spain), administered by intramuscular injection into the dorsal musculature (between the second and third dorsal spines). The tunas were maintained under natural photoperiod (24–27°C; salinity 37.5 g L^−1^; oxygen 100%) and fed with round sardinella (*Sardinella aurita*, Clupeidae), sardine (*Sardina pilchardus*, Clupeidae) and Atlantic mackerel (*Scomber scombrus*, Scombridae). A control group was handled identically but did not receive the OTC injection. All fish were individually identified using passive integrated transponder (PIT) microchips. Ethics Committee of Animal experimentation (CEEA) from the Region of Murcia code: 744/2021, register REGA ES300305440012.

Short‐term sampling was planned at 72 h post‐injection, with three individuals per dose being euthanised, while the remaining fish were sampled only in the event of natural mortality. Within the first 24 h after injection, two individuals died (one from each treatment). As tetracycline incorporates into the otolith within approximately 24 h (Pilling et al., 2007), the otoliths of these fish were also analysed. The last individual examined survived until 98 days after OTC administration.

Extracted *sagitta* otoliths were cleaned with ethanol and Milli‐Q® water, air‐dried, protected from the light to prevent the mark degradation (OTC is susceptible to sunlight irradiation) and polished (1200–3000 grit abrasive paper, 3M™ and *MicroCut*). Marks were examined using fluorescence microscopy (Leica DM LS, excitation 355–425 nm). Mark intensity was quantified with ImageJ (*Fiji package*; Schindelin et al., 2012; Rueden et al., 2017) using a customised macro that measured the mean grey value of 10 selected regions per otolith. Complementary visual assessment employed an ordinal scale (1 = low, 2 = good, 3 = excellent intensity). Differences between OTC doses were evaluated using the Mann–Whitney *U* test (*α* = 0.05), and the mark intensities are given in mean ± standard deviation (SD).

All otoliths from OTC‐treated fish displayed a clearly visible green fluorescent band under ultraviolet light (Figure 1); however, otoliths from the control group did not present green fluorescence. Quantitative analysis (Table 1) revealed a mean mark intensity of 102.2 ± 31.0 for the 100 mg kg^−1^ group and 116.8 ± 47.5 for the 200 mg kg^−1^ group, with no statistically significant differences between treatments. Although visual assessment corroborated these observations, with most otoliths classified as having ‘good’ or ‘excellent’ intensity, digital quantification (grey values obtained with Fijii/ImageJ) provides a more objective and reproducible measure. This approach, recommended by several authors (Lochet et al., 2009), allows standardised comparisons across studies and reduces the inter‐observer bias inherent to qualitative scales. Nonetheless, the variability observed in mark intensity may be influenced by biological factors, such as growth rate (Harrison & Heidinger, 1996), as well as technical factors, such as the degree of otolith polishing (Hernaman et al., 2000; Reinert et al., 1998), which should be taken into account in future research.

**FIGURE 1:**
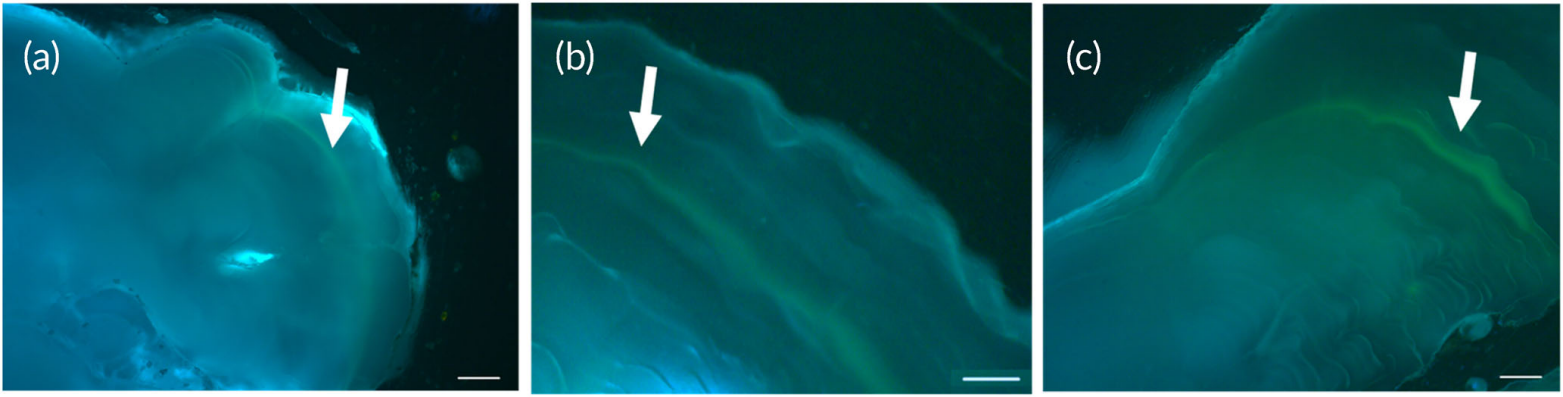
Oxytetracycline hydrochloride marks in juvenile ABFT submitted to intramuscular injection of the compound. Scale bar = 100 μm. (a) 85 dph, low intensity; (b) 80 dph, good intensity; (c) 104 dph, excellent intensity.

**TABLE 1:** Overview of juvenile ABFT injected with OTC hydrochloride, including OTC injection concentration (in milligrams per kilogram), tuna body weight (in grams), time since injection (in days), quantitative mark intensity (mean ± SD) and qualitative visibility of the mark.

OTC injection concentration (mg kg^−1^)	Tuna body weight (g)	Days post‐injection	Quantitative intensity (mean ± SD)	Qualitative intensity visibility
100	89.9	1	172.87	2
200	84.20	1	80.88	1
200	109.30	3	90.70	2
100	161.57	3	94.01	2
200	100.00	3	77.99	2
100	99.78	3	82.54	3
200	53.58	3	188.28	3
100	89.45	3	102.73	2
200	104.40	8	166.41	2
100	171.40	17	72.18	3
100	253.60	27	101.44	2
100	383.60	27	83.85	3
200	361.80	38	140.31	1
100	630.00	73	108.19	2
200	945.00	98	73.09	3

*Note:* Low visibility = 1, good = 2, excellent = 3.

These findings demonstrate that intramuscular OTC injection is a technically viable and effective method for otolith marking in juvenile ABFT. The absence of dose‐dependent differences between 100 and 200 mg kg^−1^ suggests operational flexibility for practical applications. The persistence of the fluorescent mark over 98 days of growth indicates that the method remains effective as otoliths continue to deposit calcified material. In relation to the early mortality observed in two fish that died within 24 h following injection (one per dose treatment), the results do not suggest a clear dose‐dependent toxic effect, given that: (i) no mortality was associated with a particular OTC dose; and (ii) neither significant differences in mark intensity were observed between the two concentrations tested. The short interval between treatment and mortality instead suggests that handling stress, injection trauma or individual susceptibility may have contributed, something that has been observed in other studies (Vollset et al., 2020; Warren‐Myers et al., 2018). Future studies should assess mark persistence up to commercial sizes with larger sample sizes, optimise protocols for large‐scale application and validate mark detection under commercial inspection conditions.

The relevance of this study extends beyond *T. thynnus*. Although full‐cycle aquaculture of ABFT is not yet commercially established, the successful closure of its life cycle in land‐based facilities (Campobasso et al., 2017; Ortega & De la Gándara, 2017) indicates that such systems may become viable in the near future. In this context, internal traceability, cohort identification and early growth validation will become essential tools for managing production cycles from the juvenile stages onwards. The OTC marking method evaluated here provides a simple, reproducible and directly applicable technique for these emerging systems, offering a methodological foundation that can be readily incorporated into future full‐cycle ABFT production programmes.

## AUTHOR CONTRIBUTIONS

Conceptualization: AO, DR; Data curation: ISL, DR, EB; Formal analysis: ISL, DR; Funding acquisition: AO; Investigation: ISL, EB; Methodology: AO, DR; Project administration: AO; Resources: AO, DR; Supervision: AO, DR, EB; Validation: EB; Visualization: ISL; Writing – original draft: ISL; Writing – review and editing: AO, DR, EB.

## Data Availability

The data that support the findings of this study are available from the corresponding author upon reasonable request.

## References

[jfb70516-bib-0001] Adam, S. , Stéquert, B. , & Anderson, R. C. (1996). Irregular microincrement deposition on the otoliths of skipjack tuna (*Katsuwonus pelamis*) from the Maldives. In A. A. Anganuzzi , K. A. Stobberup , & N. J. Webb (Eds.), Proceedings of the Expert Consultation on Indian Ocean Tunas, 6' Session, Colombo, Sri Lanka 25‐29 September 1995. 373. Indo‐Pacific Tuna Development and Management Programme.

[jfb70516-bib-0002] Brooks, R. C. , Heidinger, R. C. , & Kohler, C. C. (1994). Mass‐marking otoliths of larval and juvenile walleyes by immersion in oxytetracycline, calcein, or calcein blue. North American Journal of Fisheries Management, 14, 143–150. 10.1577/1548-8675(1994)014<0143:MMOOLA>2.3.CO;2

[jfb70516-bib-0003] Campobasso, F. , Fanizzi, A. , Bello, G. , Santamaria, N. , & Corriero, A. (2017). A ‘machine learning’ technique for discriminating captive‐reared from wild Atlantic bluefin tuna, *Thunnus thynnus* (Osteichthyes: Scombridae), based on differential fin spine bone resorption. Fisheries Research, 194, 42–49. 10.1016/j.fishres.2017.05.008

[jfb70516-bib-0004] Foreman, T. (1996). Estimates of age and growth, and an assessment of ageing techniques, for northern Bluefin tuna, *Thunnus thynnus*, in the Pacific Ocean. Inter‐American Tropical Tuna Commission (IATTC) Bulletin, 21(2), 75–105.

[jfb70516-bib-0005] Hallier, J. P. , Stequert, B. , Maury, O. , & Bard, F. X. (2005). Growth of bigeye tuna (*Thunnus obesus*) in the eastern Atlantic Ocean from tagging‐recapture data and otolith readings. Collective Volume of Scientific Papers, ICCAT, 57(1), 181–194.

[jfb70516-bib-0006] Harrison, D. R. , & Heidinger, R. C. (1996). Factors determining quality of Oxytetracycline Marks in fingerling walleye otoliths. Proceedings of the Annual Conference, Southeastern Association of Fish and Wildlife Agencies, 50, 174–181.

[jfb70516-bib-0007] Hernaman, V. , Munday, P. L. , & Schlappy, M. L. (2000). Validation of otolith growth‐increment periodicity in tropical gobies. Marine Biology, 137, 715–726.

[jfb70516-bib-0008] James, K. C. , Kierdorf, U. , Cooley, V. , Nikitin, V. , Stock, S. R. , & Kierdorf, H. (2024). Characterization of incremental markings in the sagittal otolith of the Pacific sardine (*Sardinops sagax*) using different imaging modalities. Minerals, 14(7), 705. 10.3390/min14070705

[jfb70516-bib-0009] Krusic‐Golub, K. , & Ailloud, L. (2022). Evaluating otolith increment deposition rates in bigeye tuna (*Thunnus obesus*) and yellowfin tuna (*T. albacares*) tagged in the Atlantic Ocean. Fishery Bulletin, 121, 1–16.

[jfb70516-bib-0010] Lochet, A. , Jatteau, P. , & Rochard, E. (2009). A reliable method to assess mark quality on fish otoliths. Fisheries Management and Ecology, 16, 508–513.

[jfb70516-bib-0011] Nagięć, M. , Czerkies, P. , Goryczko, K. , Witkowski, A. , & Murawska, E. (1995). Mass‐marking of grayling, *Thymallus thymallus* (L.), larvae by fluorochrome tagging of otoliths. Fisheries Management and Ecology, 2, 185–195. 10.1111/j.1365-2400.1995.tb00111.x

[jfb70516-bib-0012] Ortega, A. , & De la Gándara, F. (2017). Closing the life cycle of the Atlantic bluefin tuna *Thunnus thynnus* in captivity. Proceedings of Aquaculture Europe, 17, 857–858.

[jfb70516-bib-0013] Pilling, G. M. , Cotter, A. J. R. , & Metcalfe, J. D. (2007). ICCAT field manual: Chapter 4. Data for assessment and research. CEFAS.

[jfb70516-bib-0014] Reinert, T. , Wallin, J. , Griffin, M. , Conroy, M. , & VanDenAvyle, M. (1998). Long‐term retention and detection of oxytetracycline marks applied to hatchery‐reared larval striped bass, *Morone saxatilis* . Canadian Journal of Fisheries and Aquatic Sciences, 55, 539–543.

[jfb70516-bib-0015] Rueden, C. T. , Schindelin, J. , Hiner, M. C. , DeZonia, B. E. , Walter, A. E. , & Eliceiri, K. W. (2017). ImageJ2: ImageJ for the next generation of scientific image data. Bioinformatics, 18, 529. 10.1186/s12859-017-1934-z 29187165 PMC5708080

[jfb70516-bib-0016] Salvat‐Leal, I. , Ortega, A. , Blanco, E. , García, J. , & Romero, D. (2023). Elemental composition in soft tissues as a model for identifying batches of juvenile Atlantic bluefin tuna (*Thunnus thynnus*). Journal of Food Composition and Analysis, 118, 105–176.

[jfb70516-bib-0017] Sardenne, F. , Dortel, E. , Le Croizier, G. , Million, J. , Labonne, M. , Leroy, B. , Bodin, N. , & Chassot, E. (2015). Determining the age of tropical tunas in the Indian Ocean from otolith microstructures. Fisheries Research, 163, 44–57.

[jfb70516-bib-0018] Schindelin, J. , Arganda‐Carreras, I. , Frise, E. , Kaynig, V. , Longair, M. , Pietzsch, T. , Rueden, C. , Saafeld, S. , Schmid, B. , Tinevez, J. Y. , White, D. J. , Hartenstein, V. , Eliceiri, K. , Tomancak, P. , & Cardona, A. (2012). Fiji: An open‐source platform for biological‐image analysis. Nature Methods, 9, 676–682. 10.1038/nmeth.2019 22743772 PMC3855844

[jfb70516-bib-0019] Vollset, K. W. , Lennox, R. J. , Thorstad, E. B. , Auer, S. , Bär, K. , Larsen, M. H. , Mahlum, S. , Näslund, J. , Stryhn, H. , & Dohoo, I. (2020). Systematic review and meta‐analysis of PIT tagging effects on mortality and growth of juvenile salmonids. Reviews in Fish Biology and Fisheries, 30, 553–568. 10.1007/s11160-020-09611-1

[jfb70516-bib-0020] Warren‐Myers, F. , Dempster, T. , & Swearer, S. E. (2018). Otolith mass marking techniques for aquaculture and restocking: Benefits and limitations. Reviews in Fish Biology and Fisheries, 28, 485–501. 10.1007/s11160-018-9515-4

[jfb70516-bib-0021] Weber, D. D. , & Ridgway, G. J. (1962). The deposition of tetracycline drugs in bones and scales of fish and its possible use for marking. The Progressive Fish‐Culturist, 24(4), 150–155.

[jfb70516-bib-0022] Weber, D. D. , & Ridgway, G. J. (1967). Marking Pacific salmon with tetracycline antibiotics. Journal of the Fisheries Research Board of Canada, 24, 849–865.

[jfb70516-bib-0023] Wild, A. , & Foreman, T. J. (1980). The relationship between otolith increments and time for yellowfin and skipjack tuna marked with tetracycline. Inter‐American Tropical Tuna Commission Bulletin, 17(7), 509–541.

